# Is there a role for lung surgery in initially unresectable non-small cell lung cancer after tyrosine kinase inhibitor treatment?

**DOI:** 10.1186/s12957-022-02833-6

**Published:** 2022-11-26

**Authors:** Nguk Chai Diong, Chia-Chuan Liu, Chih-Shiun Shih, Mau-Ching Wu, Chun-Jen Huang, Chen-Fang Hung

**Affiliations:** 1 Division of Thoracic Surgery, Department of Surgery, Kuala Lumpur General Hospital, Kuala Lumpur, Malaysia; 2 Division of Thoracic Surgery, Department of Surgery, Koo Foundation Sun Yat-Sen Cancer Center, 125, Lide Road, Beitou District, Taipei, 11259 Taiwan; 3 Department of Medical Oncology, Koo Foundation Sun Yat-Sen Cancer Center, Taipei, Taiwan; 4 Department of Pulmonary Medicine and Intensive Care Medicine, Koo Foundation Sun Yat-Sen Cancer Center, Taipei, Taiwan; 5 Department of Research, Koo Foundation Sun Yat-Sen Cancer Center, Taipei, Taiwan

**Keywords:** Epidermal growth factor receptor (EGFR), Molecular targeted therapy, Non-small cell lung cancer (NSCLC), VATS

## Abstract

**Background:**

The role of lung surgery in initially unresectable non-small cell lung cancer (NSCLC) after tyrosine kinase inhibitor (TKI) treatment remains unclear. We aimed to assess the survival benefits of patients who underwent surgery for regressed or regrown tumors after receiving TKI treatment.

**Methods:**

The details of patients diagnosed with unresectable NSCLC treated with TKI followed by lung resection from 2010 to 2020 were retrieved from our database. The primary endpoint was 3-year overall survival (OS), whereas the secondary endpoints were a 2-year progression-free survival (PFS), feasibility, and the safety of pulmonary resection. The statistical tests used were Fisher’s exact test, Kruskal Wallis test, Kaplan-Meier method, Cox proportional hazards model, and Firth correction.

**Results:**

Nineteen out of thirty-two patients were selected for the study. The patients underwent lung surgery after confirmed tumor regression (17 [89.5%]) and regrowth (two [10.5%]). All surgeries were performed via video-assisted thoracoscopic surgery: 14 (73.7%) lobectomies and five (26.3%) sublobar resections after a median duration of 5 months of TKI. Two (10.5%) postoperative complications and no 30-day postoperative mortality were observed. The median postoperative follow-up was 22 months.

The 2-year PFS and 3-year OS rates were 43.9% and 61.5%, respectively. Patients who underwent surgery for regressed disease showed a significantly better OS than for regrowth disease (HR=0.086, 95% CI 0.008–0.957, *p*=0.046). TKI-adjuvant demonstrated a better PFS than non-TKI adjuvant (HR=0.146, 95% CI 0.027–0.782, *p*=0.025).

**Conclusion:**

Lung surgery after TKI treatment is feasible and safe and prolongs survival via local control and directed consequential therapy. Lung surgery should be adopted in multimodality therapy for initially unresectable NSCLC.

## Background

Lung cancer is the top-ranked cancer-related cause of death worldwide [1]. Approximately 85% of lung cancers are non-small cell lung cancers (NSCLCs). More than 70% of these cases are diagnosed at stages III and IV [2], which are mainly unresectable. The definition of “unresectable” in lung cancer is subjective, and consensus on its definition is currently lacking [3]. Generally, unresectable NSCLC is represented by stage III NSCLC, in which the tumor is >7 cm in diameter or invades adjacent structures (T4), multistations, or bulky mediastinal lymph nodes (N2-3), and stage IV disease, in which distant metastasis (M1) is detected. Conventionally, the treatment for unresectable stage III NSCLC is concurrent platinum-based doublet chemotherapy and radiotherapy (CCRT), whereas for stage IV NSCLC is chemotherapy. These treatments yield low 5-year overall survival (OS) rates of 15% and 0–10% for stage III and stage IV disease, respectively [4, 5].

In the last two decades, significant advancements in molecular-targeted therapy have caused a paradigm shift in the standard treatment of metastatic NSCLC. Tyrosine kinase inhibitors (TKI), molecular-targeted drugs, inhibit specific oncogenic drivers, such as the epidermal growth factor receptor (EGFR) and anaplastic lymphoma kinase (ALK), which trigger cancer evolution. Multiple randomized trials have demonstrated the superiority of TKI treatment in stage IV NSCLC, with a better objective response rate (ORR, 60–80%), progression-free survival (PFS, 9–13 months), and toxicity safety profile than standard chemotherapy [6–11]. Hence, the American Society of Clinical Oncology, European Society for Medical Oncology, and National Comprehensive Cancer Network adopted EGFR-TKI and ALK-TKI as first-line therapies for EGFR- and ALK-positive advanced NSCLC, respectively [12–14].

The main drawback of TKI therapy is disease progression within approximately 1 year of administration because of acquired resistance [6–11], limiting its long-term use. During TKI therapy, the tumor may respond by shrinking to residual disease or remain stable to regrow. Consensus on the role of surgical resection for these lesions is lacking.

The role of TKI as neoadjuvant therapy remains unclear. Because of its low toxicity profile and rapid and outstanding ORR, TKI may help downstage unresectable stage III NSCLC to a resectable state in a favorable duration while maintaining the patient’s performance status.

We aimed to evaluate the effect of lung surgery on residual or regrowth lesions after response to TKI therapy in initially unresectable NSCLC patients by assessing the survival rate and the feasibility and safety of the surgery.

## Methods

### Study design and patients

We retrieved the records of all patients diagnosed with NSCLC who underwent lung resection after TKI therapy from our hospital database between January 2010 and December 2020. The inclusion criteria were patients diagnosed with unresectable NSCLC, TKI treatment followed by lung surgery, and no previous lung surgery before TKI therapy. We accepted patients who received chemotherapy, radiotherapy, or both before TKI therapy.

Clinical staging was established according to the eighth edition of the American Joint Committee on Cancer TNM with chest computed tomography (CT) for pulmonary lesions, brain magnetic resonance imaging to exclude brain metastasis and positron emission tomography–CT for nodal involvement and presence of distant metastasis.

An unresectable tumor was defined as a tumor exceeding a 7-cm diameter or direct tumor invasion into adjacent structures (T4), where resection may be deemed incomplete or in the N2-3 state for stage III NSCLC and distant metastasis for stage IV NSCLC. TKI was administered as neoadjuvant therapy for unresectable stage III NSCLC, for which radical resection was expected after the therapy. Patients with stage IV NSCLC were treated with TKI for which lung resection was considered only when the disease was converted to localized disease.

All treatment decisions were made based on tumor board discussion. Patients received TKI monotherapy based on the status of the molecular alterations: EGFR mutation with EGFR-TKI and ALK translocation with ALK-TKI. These patients were followed up with chest CT every 1 to 3 months to evaluate the response to TKI in the pulmonary and metastatic lesions according to the response evaluation criteria for solid tumors (RECIST 1.1).

Tumor regression was defined as a reduction of >30% in the size of the primary tumor after TKI treatment compared to the CT imaging size at diagnosis. Tumor regrowth was defined as an increase of >20% in the size of the primary tumor despite ongoing TKI treatment.

The feasibility and safety of pulmonary surgery were assessed based on surgery type and approach, the number of lymph nodes dissected, completeness of surgical resection, surgery duration, intraoperative blood loss, postoperative complications, and 30-day postoperative mortality. Patients with peripheral tumor and size <2 cm in diameter were subjected to sublobar resection. Otherwise, standard lobectomy and systemic lymphadenectomy were performed.

Postoperatively, adjuvant therapy was provided based on the resected pathology results. Surveillance was conducted every 3 months with chest CT for the first 3 years, followed by surveillance every 6 months.

The Institutional Review Board approved the study (IRB number 20220117A, dated 24 January 2022) and waived the requirement for informed consent from the patients.

### Endpoints

The primary endpoint was the 3-year OS after lung surgery. The secondary endpoints were 2-year PFS and feasibility and the safety of pulmonary surgery after TKI treatment.

### Statistical analysis

PFS was calculated from the surgery date to the date of disease relapse, whereas OS was calculated from the date of surgery to the date of patient death or last evaluation. Differences in demographics and clinical characteristics were performed using Fisher’s exact test for categorical data and Kruskal-Wallis test for continuous data. The Saphiro-Wilk test was used to test the normality of the data. The Cox proportional hazards model and Firth correction method were used to calculate the hazard ratio (HR) and corresponding 95% confidence intervals (CIs). A *p*<0.05 was considered statistically significant. Survival curves were estimated using the Kaplan–Meier method and compared with log-rank test curves. Statistical analyses were performed using the SAS software (v.9.4; SAS Institute, Inc., Cary, NC).

## Results

### Patient and clinical characteristics

**Fig. 1:**
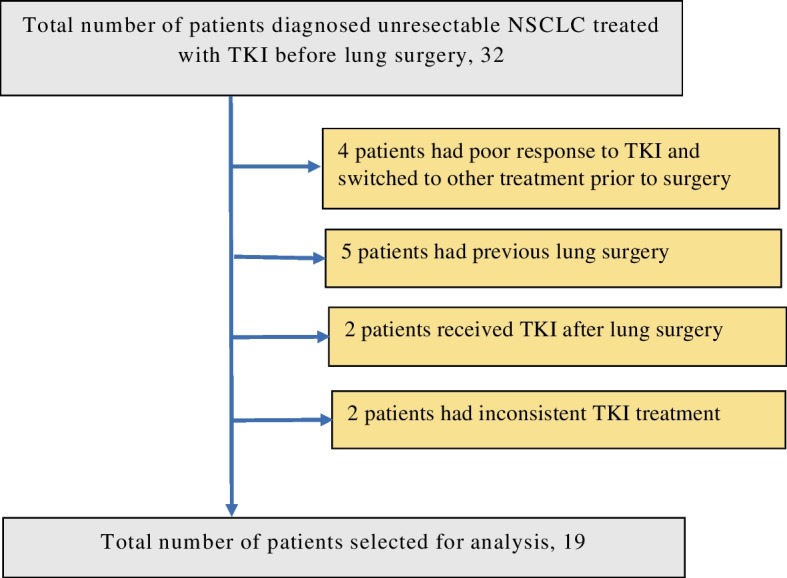
Flow chart for case selection. NSCLC, non-small cell lung cancer; TKI, tyrosine kinase inhibitor; PFS, progression-free survival; OS, overall survival

Among the 32 patients enrolled, five previously had lung surgery, four exhibited a poor response to TKI and switched to other therapies before the lung surgery, two received TKI after lung surgery, and two received inconsistent TKI treatment. Nineteen out of the thirty-two patients were selected for analysis (Fig. 1).

The demographic data and clinical characteristics of all enrolled patients are listed in Table 1. All but one patient with stage III squamous cell carcinoma (SCC) were confirmed to have adenocarcinoma and tested for EGFR mutation and ALK rearrangement at diagnosis. This one patient was subjected to EGFR-TKI (erlotinib) after failed definitive chemotherapy and radiotherapy and responded with stable disease for 5 years, at which point the tumor started to regrow. The patient underwent salvage pulmonary resection, and the pathology revealed adenosquamous carcinoma and wild-type for EGFR and ALK.

**Table 1:** Demographics and clinical characteristics of patients

Variable	*N*=19 (%)/*[range]*	*P* value
Gender		0.326
Female	12 (63.2)	
Male	7 (36.8	
Age, median	58 *[34–70]*	
ECOG status		0.617
0	13 (68.4)	
1	6 (31.6)	
Histopathology (biopsy)		0.368
Adenocarcinoma	18 (94.7)	
Squamous cell carcinoma	1 (5.3)	
Genetic mutation		0.282
EGFR	16 (84.2)	
ALK	2 (10.5)	
Nil	1 (5.3)	
Clinical stage at diagnosis		0.617
IIIA/IIIB	4/2 (31.6)	
IVA/IVB	12/1 (68.4)	
Preoperative staging after TKI		0.154
IA	2 (10.5)	
IIA/IIB	2/2 (21.1)	
IIIA	13 (68.4)	
ypStage		0.352
IA/IB	4/3 (36.8)	
IIB	2 (10.5)	
IIIA/IIIB	7/1 (42.1)	
IVA	2 (10.5)	
Type of preoperative TKI		0.091
Afatinib	7 (36.8)	
Gefitinib	6 (31.6)	
Erlotinib	3 (15.8)	
Osimertinib	1 (5.3)	
Alectinib	1 (5.3)	
Crizotinib	1 (5.3)	
Median duration of preoperative TKI, months	5 [0.8–66]	0.611
Neoadjuvant	2.5 [*1.5-6*]	
Treatment	5.8 [*0.8-66*]	
Indication for preoperative TKI		1.000
Neoadjuvant	5 (26.3)	
Treatment	14 (73.7)	
Indication for surgery		0.123
Regressed disease	17 (89.5)	
Regrowth disease	2 (10.5)	
Duration of follow-up, median months	22 [*1–85*]	

*ECOG* Eastern Cooperative Oncology Group, *EGFR* epidermal growth factor receptor, *ALK* anaplastic lymphoma kinase, *TKI* tyrosine kinase inhibitor

The median duration of preoperative TKI administration was 5 months (0.8–66.0 months). Indications for surgery included regressed disease in 17 (89.5%) cases and tumor regrowth in two (10.5%) cases.

### Safety of surgery

All patients underwent video-assisted thoracoscopic surgery (VATS), with lobectomy performed in 14 (73.7%) patients and sublobar resection in five (26.3%) patients (Table 2). Seventeen out of nineteen patients (89.5%) had complete tumor resection (R0), and two (10.5%) had R2 resection. Two patients (10.5%) developed postoperative complications: bleeding and thoracic empyema. Both complications were managed by VATS evacuation of clots and decortication. No postoperative mortality was reported within 30 days.

**Table 2:** Surgery, perioperative, and postoperative outcomes

Variable	*n* (%)/[*range*]	*P* value
Surgery, (VATS)		0.603
Lobectomy	14 (73.7)	
*n= RUL/RML/RLL/LUL/LLL*	*3/1/5/2/3*	
Sublobar resection	5 (26.3)	
*n=Trisegmentectomy/lingulectomy/subsegmentectomy/Wedge resection*	*2/1/1/1*	
Number of lymphadenectomy, median	20 *[5-38]*	
Complete resection		1.000
R0	17 (89.5)	
R2	2 (10.5)	
Duration of surgery, median minutes	180 *[60-380]*	0.020
Blood loss, median ml	40 *[10-550]*	0.049
Postoperative complications		0.614
Bleeding	1 (5.3)	
Thoracic empyema	1 (5.3)	
Nil	17 (89.5)	
30-day postoperative mortality	0 (0)	
Median time to relapse, months	13 *[6-41]*	
Relapse		<0.001
Alive without relapse	10 (52.6)	
Alive with relapse	2 (10.5)	
Died with relapse	7 (36.8)	
T790M status		
Positive	1 (5.3)	
Negative	1 (5.3)	
Unknown/not done	17 (89.4)	
Recurrence pattern		<0.001
Intrathoracic recurrence/metastases	2 (10.5)	
Distant metastases	3 (15.8)	
Intrathoracic and distant metastases	4 (21.1)	
Nil	10 (52.6)	
Postoperative adjuvant therapy		0.129
TKI	13 (68.4)	
Non-TKI	6 (31.6)	

*VATS* video-assisted thoracoscopic surgery, *RUL* right upper lobe, *RML* right middle lobe, *RLL* right lower lobe, *LUL* left upper lobe, *LLL* left lower lobe, *TKI* tyrosine-kinase inhibitor

### Outcomes after surgery

Postoperatively, 13 (68.4%) patients continued with the same TKI therapy, except one who switched to osimertinib because of a positive T790M mutation from the specimen (Table 2). The remaining patients received adjuvant chemotherapy, radiotherapy, or both. Nine (47.4%) patients developed relapse at a median duration of 13 months (6–41 months): intrathoracic recurrence or metastases, two (10.5%); distant metastases, three (15.8%); and both, four (21.1%). The median duration of postoperative follow-up was 22 months (1–85 months).

**Fig. 2:**
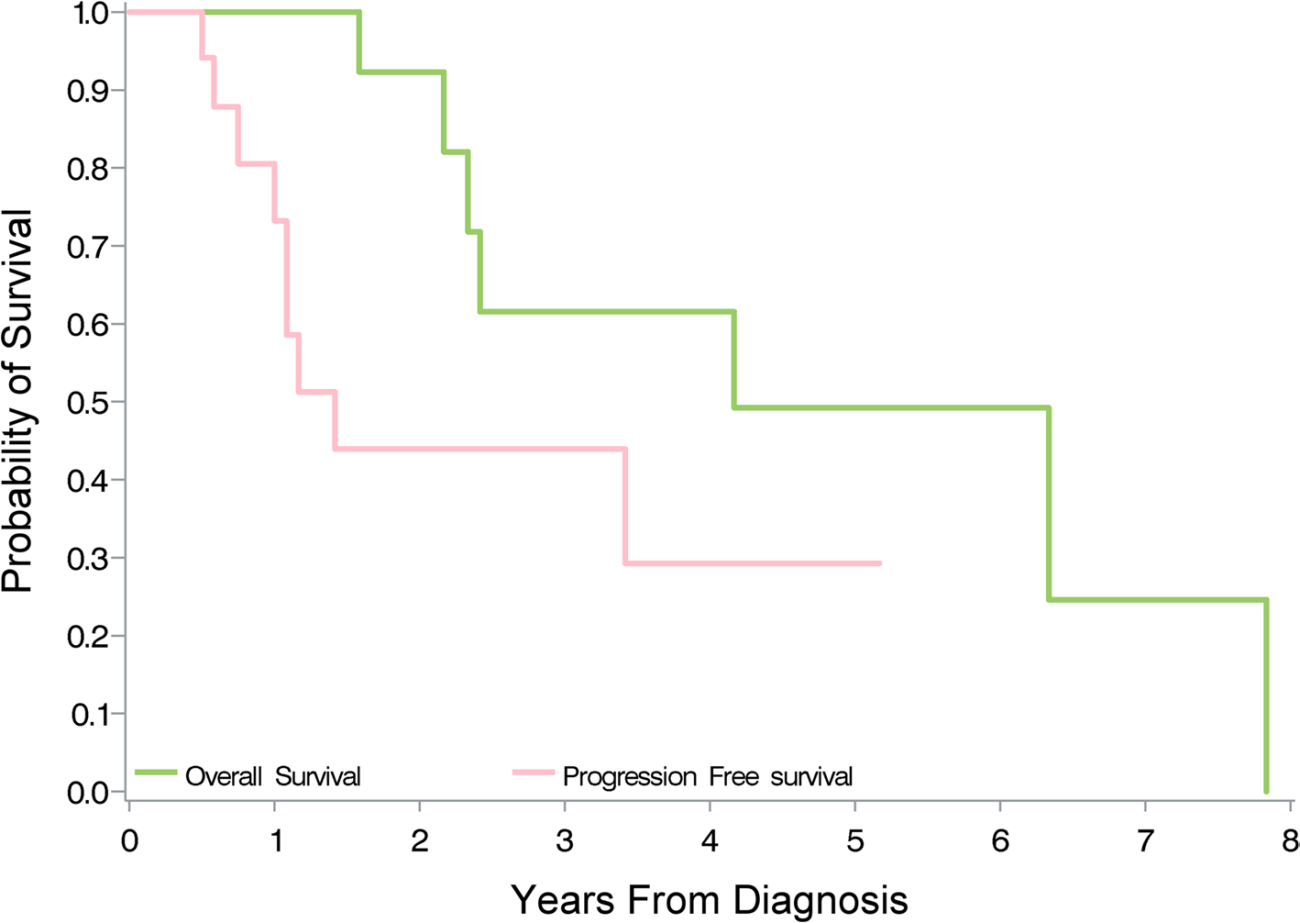
The 3-year overall survival was 61.5% and the 2-year progression-free survival was 43.9%

The 2-year PFS and 3-year OS rates were 43.9% and 61.5%, respectively (Table 3 and Fig. 2), with median PFS and OS values of 17 and 50 months, respectively.

**Table 3:** Factors associated with overall survival and progression-free survival

Variable	Overall survival			Progression-free survival		
	HR	95% CI	***p***	HR	95% CI	***p***
Gender Female vs male	0.927	0.154–5.563	0.934	0.424	0.103–1.739	0.234
ECOG 0 vs 1	5.497	0.542–66.801	0.150	0.873	0.217–3.511	0.849
Histopathology Adenocarcinoma vs SCC	0.087	0.005–1.393	0.084	0.232	0.024–2.250	0.208
Genetic mutation EGFR vs ALK	0.580	0.015–39.800	0.277	2.003	0.243–260.278	0.657
Clinical stage III vs IV	0.853	0.152–4.794	0.857	1.632	0.437–6.095	0.466
Preoperative stage I/II vs III	3.205	0.582–17.663	0.181	4.377	1.083–17.691	*0.038*
ypStage I/II/III vs IV	0.207	0.018–2.320	0.202	0.221	0.062–1.900	0.344
Indication for TKI Neoadjuvant vs treatment	0.375	0.043–3.287	0.376	1.184	0.294–4.764	0.812
Indication for surgery Regressed vs regrowth	0.086	0.008–0.957	*0.046*	0.299	0.054–1.652	0.166
Surgery Lobectomy vs sublobectomy	0.348	0.031–3.853	0.289	0.904	0.179–4.566	0.903
Completeness resection R0 vs R2	4.828	0.431–54.077	0.202	2.911	0.526–16.100	0.221
Postoperative TKI adjuvant Yes vs no	0.506	0.099–2.587	0.413	0.146	0.027–0.782	*0.025*
Relapse Yes vs no	6.268	0.701–825.479	0.259			

*ECOG* Eastern Cooperative Oncology Group, *EGFR* epidermal growth factor receptor, *ALK* anaplastic lymphoma kinase, *SCC* squamous cell carcinoma, *TKI* tyrosine kinase inhibitor, *HR* hazard ratio, *CI* confidence interval

### Prognostic factors of PFS and OS after lung surgery

Univariate analysis identified patients who underwent surgery for regressed disease was associated with significant better OS than regrowth disease (HR=0.086, 95% CI 0.008–0.957, *p*=0.046) and adjuvant TKI treatment showed better PFS than non-TKI adjuvant treatment (HR=0.146, 95% CI 0.027–0.782, *p*=0.025). Preoperative stage I/II after TKI treatment showed poorer PFS than stage III disease (HR 4.377, CI 95% 1.083–17.691, *p*=0.038) (Table 3).

## Discussion

The observed 3-year OS of 61.5% with a median OS of 50 months from lung surgery after TKI therapy for initially unresectable stage III and IV NSCLC is encouraging. This value generally exceeded the 3-year OS and a median OS of stages III and IV, 7–55%, and 6–29 months, respectively [5, 15]. In addition to the efficacy of TKI therapy, the contribution of lung resection should be highlighted to achieve this milestone.

The development of molecular-targeted therapy has allowed patients to gain extraordinary responses to treatment with less toxicity. Therefore, advanced NSCLC patients are expected to live longer with improved treatment options and maintained Eastern Cooperative Oncology Group (ECOG) status, particularly in oligometastatic NSCLC, as observed in our cohort. Oligometastasis is defined as five or fewer metastases that involve three or fewer organs [16] and has a better prognosis than the general stage IV cohort, with a 5-year OS of 30 vs. 0–10% [5, 17, 18]. Hence, oligometastatic NSCLC deserves aggressive treatment, including primary lung resection.

Gomez et al. demonstrated comprehensive local consolidation therapy (LCT) after systemic therapy in which local therapy applied to primary and metastatic lesions of NSCLC significantly prolonged PFS and OS than maintenance therapy and observation: PFS was 14.2 vs. 4.4 months and OS was 41.2 vs. 17.7 months for LCT vs. maintenance therapy, respectively [19]. Mitchell et al. compared the LCT for primary lung lesions treated with surgery to radiotherapy for oligometastatic NSCLC and observed superior 5-year OS in the surgery group (48.0 vs. 24.2%) [20]. Compared to radiation and ablation therapy, surgery has the following advantages: obtaining a valuable specimen for the complete assessment of tumor response to treatment, exploring the resistance mechanism, evaluating actionable biomarkers to direct subsequent treatment, overcoming acquired resistance, reducing the amount of circulating tumor cells and tumor heterogeneity, and enhancing the subsequent systemic therapy effect [21, 22].

Evidence for the benefit of lung surgery after TKI therapy is scarce, as shown by the limited number of patients reported in most published papers (Table 4) [23–26]. The median PFS and OS values ranged from 10 to 15 and 17 to 68 months, respectively. Compared to previous studies, our median PFS was higher (17 months), whereas our median OS was within the previously reported range. Pulmonary resection after TKI therapy is technically challenging because of the dense scar tissue formed at the pleura and hilum as a sequela of the tissue reaction to the treatment. Consequently, extended operating hours and postoperative morbidity are anticipated. The complication and mortality rates observed in our study are compatible with those reported for VATS lobectomy (10.5 vs. 10–20% and 0 vs. 0–2%, respectively) [27, 28]. The favorable perioperative outcome was attributed to the good ECOG status, low toxicity profile of TKI, case selection, improvements in perioperative care, prevention of pneumonectomy, and minimally invasive surgery. Overall, lung surgery after TKI is feasible and safe with appropriate patient selection.

**Table 4:** Comparing our results with other studies

Study	*n*	Duration of TKI, m	Clinical stage, *n* (%)	Surgery, *n* (%)	Post operative complications, *n*	Adjuvant TKI, Y/N/U	PFS/OS, m
Ohtaki et al. [23]	36	14	IIIA, 8 (22.2) IIIB, 4 (11.1) IV, 21 (58.3) R, 3 (8.3)	Lobectomy, 28 (77.8) Segmentectomy, 3 (8.3) Wedge resection, 2 (5.6) Bilobectomy, 1 (2.8) Pneumonectomy, 2 (5.6)	Bleeding, 1 Empyema, 1	16/20/0	15/58
Song et al. [24]	9	6 (2–46)	IIIA, 2 (22.2) IIIB, 1 (11.1) IV, 6 (66.7)	Lobectomy, 9 (100%)	Atrial fibrillation, 1	9/0/0	14/17
Yamamoto et al. [25]	24	3 (0.7–36)	IB, 1 (4.2) IIIA, 8 (33.3) IIIB, 5 (20.8) IV, 10 (41.7)	Lobectomy, 15 (62.5) Bilobectomy, 5 (20.8) Pneumonectomy, 4 (16.7)	Empyema, 1 Chylothorax, 1	12/9/3	10/68
Ning et al. [26]	10	3 (3–5)	IIIA, 2 (20) IIIB, 8 (80)	Lobectomy, 9 (90) Pneumonectomy, 1 (10)	Death, 1	9/0/0	14/36

*CR* complete response, *TKI* tyrosine kinase inhibitor, *PFS* progression-free survival, *OS* overall survival, *R* recurrent, *U* unknown, *m* month, *Y* yes, *N* No

The National Health Insurance of Taiwan approved the prescription of osimertinib, a third-generation EGFR-TKI, as the treatment for acquired resistance to the EGFR T790M mutation at the end of the study period in 2020. Before then, the T790M test was not widely performed. As only one patient in the cohort tested T790M-positive, we could not evaluate the benefit of surgery for acquired resistance compared to the osimertinib treatment alone.

Several studies have shown that regressed disease is a better predictor for favorable OS and PFS than stable disease [29, 30]. Although our study revealed that patients with regressed disease had a better OS than regrowth disease, the disparity between regressed and regrowth diseases (89.5% and 10.5%, respectively) in a small study cohort made the comparison tenuous. Hence, a study with large cohorts is required to validate the findings.

Five patients with unresectable stage III NSCLC at diagnosis received TKI as neoadjuvant therapy for a median duration of 2.5 months, followed by radical resection. All achieved R0 resection. The 3-year OS showed superior results compared to the PACIFIC trial, in which a similar group of patients received durvalumab after standard CCRT therapy (61.5 vs. 56.7%) [31]. Wu et al. reported median and maximum time to response values of 1.7 and 3.9 months, respectively [29]. Takeda et al. reported a median time to response of 1 month with most patients achieving complete response and a partial response within 2 months [30]. We deduced that TKI is safe and effective for 1–4 months of administration as a neoadjuvant before undergoing radical resection.

Evidence of TKI as adjuvant therapy is rare. Riely et al. advocated the continuation of TKI after failed TKI treatment because some tumor cells remaining sensitive to EGFR inhibition [32]. These issues should all be resolved after complete resection, and logically, TKI should be discontinued. However, our study showed that TKI continuation after the lung surgery significantly prolonged PFS compared to non-TKI adjuvant therapy. TKI is believed to act as cytostatic instead of cytotoxic drug, and it cannot eliminate micrometastatic cancer cells [33]. Hence, the continuation of TKI after surgery helps control subclinical micrometastatic cancer cells. The ADAURA trial studied osimertinib as adjuvant therapy for resectable stages IB–IIIA EGFR-mutant NSCLC and showed a significantly longer 2-year disease-free survival than with placebo (90 vs 44%) [34]. These results clarified the role of TKI as adjuvant therapy, and further research is justified.

The limitations of this study include the following: it was a retrospective, single-arm investigation conducted at a single center with a small sample size, short follow-up, highly selected patients, and heterogeneity of patient characteristics and clinical course. All stage IV patients were clinically downstaged to M0 before surgery. All regrowth tumors exhibited solitary progression. Only patients with a favorable response to TKI treatment were recruited, and those with an inadequate response were excluded. These factors all lead to a better prognosis. A prospective study with a larger sample size and a nonsurgical control group for comparison is required to validate the role of lung surgery in this context.

Lung surgery for initially unresectable NSCLC after TKI therapy is feasible and safe, helps to solve the problem of acquired resistance, and prolongs survival by local control and directed consequential treatment via surgical specimen analysis. Surgery should be considered a part of multimodality treatment, particularly for patients with an excellent response to TKI treatment.

## Data Availability

All data generated or analyzed during this study are included in this published article.

## Abbreviations

NSCLC: Non-small cell lung cancer
TKI: Tyrosine kinase inhibitor
EGFR: Epidermal growth factor receptor
ALK: Anaplastic lymphoma kinase
OS: Overall survival
PFS: Progression-free survival
ORR: Objective response rate
VATS: Video-assisted thoracoscopic surgery
